# Serum microRNA-4297 is a sex-specific predictive biomarker of glioma grade and prognosis

**DOI:** 10.3389/fneur.2022.888221

**Published:** 2022-07-27

**Authors:** Wenshen Xu, Liming Huang, Bingsen Xie, Bin Yang

**Affiliations:** ^1^Department of Laboratory Medicine, Gene Diagnosis Research Center, The First Affiliated Hospital, Fujian Medical University, Fuzhou, China; ^2^Fujian Key Laboratory of Laboratory Medicine, The First Affiliated Hospital, Fujian Medical University, Fuzhou, China; ^3^Department of Oncology, The First Affiliated Hospital, Fujian Medical University, Fuzhou, China; ^4^Molecular Oncology Research Institute, The First Affiliated Hospital, Fujian Medical University, Fuzhou, China; ^5^Department of Neurosurgery, The First Affiliated Hospital, Fujian Medical University, Fuzhou, China; ^6^Department of Neurosurgery, National Regional Medical Center, Binhai Campus of the First Affiliated Hospital, Fujian Medical University, Fuzhou, China; ^7^Fujian Institute of Neurology, The First Affiliated Hospital, Fujian Medical University, Fuzhou, China

**Keywords:** glioma, miR-4297, MGMT, sexual dimorphism, biomarker

## Abstract

**Background:**

Gliomas account for nearly 80% of brain cancers, tending to occur more frequently in men with adverse outcomes. Emerging microRNAs have been positioned as promising predictors for glioma's histological grade and prognosis. However, there have been few studies concerning the sex-biased impacts on the clinical approach for the potential microRNA-4297 (miR-4297).

**Methods:**

We utilized GSE139031micro-RNAs profiling to analyze serum miR-4297 expression in glioma. A total of 114 newly diagnosed glioma patients at the First Affiliated Hospital of Fujian Medical University from January 2017 to February 2021 were recruited and prospectively followed up. The association of miR-4297 levels with glioma grade and prognosis was investigated. Luciferase reporter gene assays and genotype analyses were carried out to explore the potential mechanism of sexually dimorphic miR-4297 in glioma.

**Results:**

Serum miR-4297 levels were notably down-regulated in glioma. Besides, serum miR-4297 levels were positively associated with the high grades, which were exclusively present for females. The positive correlations of miR-4297 with O6-methylguanine-DNA methyltransferase (MGMT) protein and mean platelet volume were also observed in females. IDH-mutant females had decreased miR-4297. Median PFS time for females with miR-4297 ≥ 1.392 was distinctly shorter than those with miR-4297 <1.392 (12.3 months vs. 42.89 months, *p* = 0.0289). Based on multivariate logistic regression, miR-4297-based equation model was established as FHGRS. AU-ROC analysis revealed FHGRS exhibited a robust performance in predicting high-grade glioma in females (*p* < 0.001), whereas there was no such relationship in males. Furthermore, the MGMT-3'UTR variant rs7896488 in the specific binding region of miR-4297 was correlated with prognosis.

**Conclusion:**

Our study uncovers sex-dependent characterization of serum miR-4297 in predicting glioma grade and the relapse risk for female patients, which underscores the clinical benefits of sex-specific analysis in non-coding RNA research.

## Introduction

Glioma, originating from glial cells, is the most frequent malignant primary intracranial neoplasm, characterized by a highly aggressive phenotype, high recurrence rate, and high mortality. Gliomas are classified from grade I to grade IV according to the World Health Organization (WHO) grading criteria of primary brain tumors (1). As estimated in Cancer Statistics 2021 (2), brain and other nervous system tumors are still the leading cause of cancer death among men aged < 40 years and women aged < 20 years. China was one of the three countries with the largest incident cases and the most deaths of CNS cancer (3). Despite the considerable progress in the management of glioma, the prognosis is universally poor, especially for high-grade gliomas (HGGs: WHO grade III–IV) (1), where tumor cell heterogeneity and sex differences influence clinical outcomes.

To date, glioma diagnosis and prognostic molecular markers are obtained from neurosurgical biopsy. Nevertheless, tissue biopsy cannot achieve early diagnosis, targeted treatment, and dynamic monitoring. Hence, there is an urgent need to identify accurate, convenient and non-invasive biomarkers for glioma diagnosis and prognosis. MicroRNAs (miRNAs) are a class of single-stranded small RNA regulatory molecules, which remain stable for a relatively long period in human biofluids (4, 5). Deregulation of miRNA activity has been demonstrated in the initiation, progression, and metastasis of human cancers (6). MiRNAs-based glioma research have been extensively carried out throughout the last decade. There has been a wealth of data implicating blood-sample-based miRNAs would be promising markers for glioma grades classification and prognostic evaluation (7–9). However, to the best of our knowledge, there is no definite application of miRNAs for glioma clinical management. It seems that the comprehensive utilization of blood-sampled-miRNAs still faces challenges (10, 11).

Emerging evidences have indicated that gliomas with the same histopathological type also exhibit distinct symptoms with varying prognostications. Biological heterogeneity has always been the conundrum to improve glioma treatments (12). Epidemiological data have demonstrated that the overall incidence of glioma are higher in males with the adverse outcomes (13). In addition, it has been reported that O6-methylguanine-DNA methyltransferase (MGMT) promoter methylation, which has been regarded as the indicator of temozolomide (TMZ) chemotherapy resistance, is also more likely to occur in glioma female patients (14). Hence, sex heterogeneous effects on glioma tumorigenesis and prognosis should not be neglected.

In previous studies, researchers have noticed that miRNAs have sex-specific roles in chronic kidney disease, cardiovascular disease, and neurodegenerative disease (15–17). It has been reported that microRNA expressions differ between males and females and the loss of microRNAs leads to sex-specific changes in the presence, development, and progress of the diseases (18). Therefore, with regards to miRNA-related application evaluation, failing to take the potential disparities between males and females into consideration could lead to a sex bias. Owing to the limited study concerning the sex impact on miRNA-oriented clinical utilization for glioma patients, we aimed to provide novel insights into the predictive roles of miR-4297 for glioma grade and prognostication in a sex-dependent manner.

## Materials and methods

### Study population

**Figure 1:**
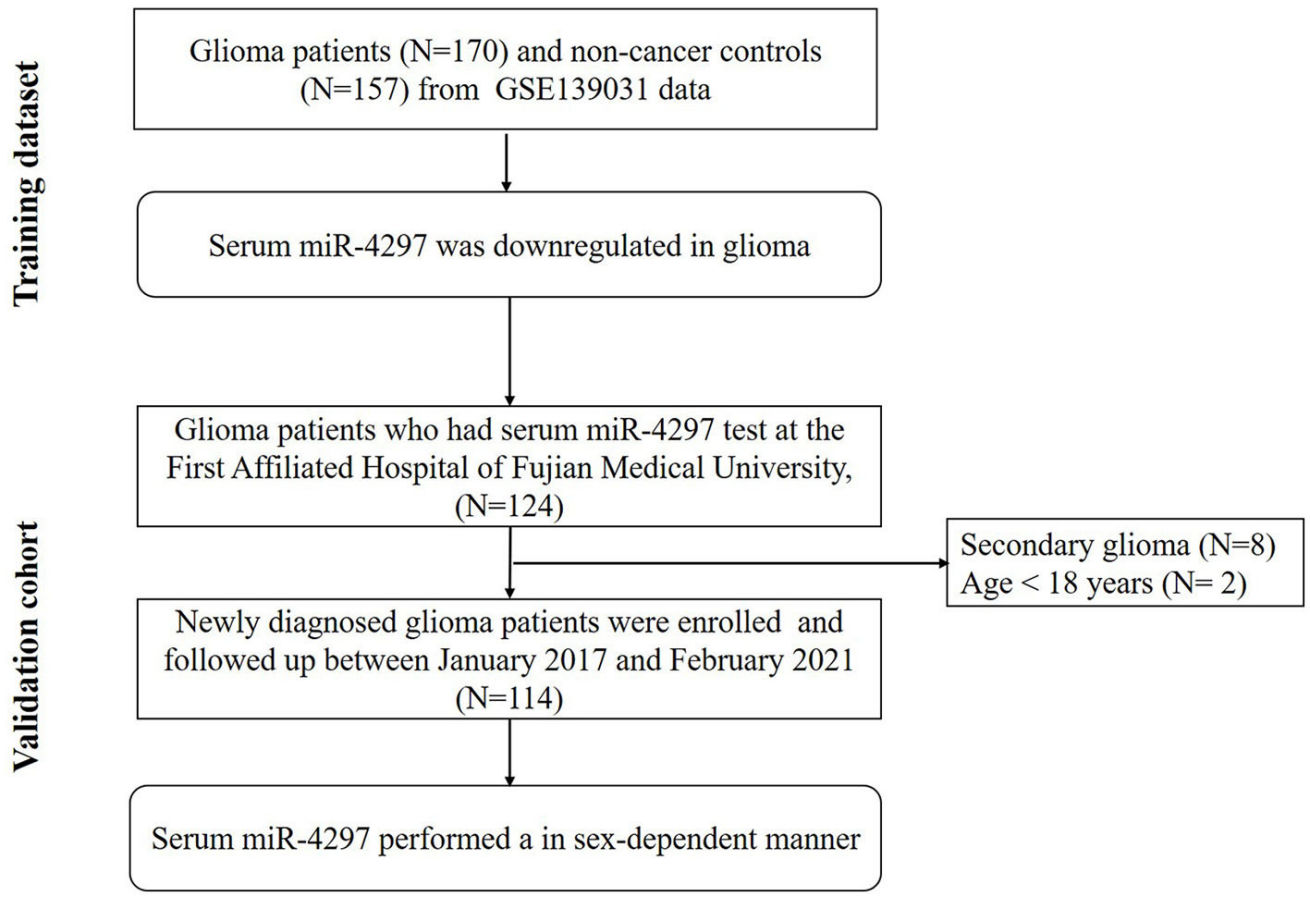
Flow chart describing the process for exploring the sex-specific role of miR-4297.

The study flow chart was present in Figure 1. Serum miR-4297 expression dataset were downloaded from serum non-coding RNA profiling by array (GSE139031, https://www.ncbi.nlm.nih.gov/geo/query/acc.cgi?acc=GSE139031, updated to Dec 31, 2019) in the Gene Expression Omnibus (GEO, http://www.ncbi.nlm.nih.gov/geo/). Based on GPL21263 (3D-Gene Human miRNA V21_1.0.0) by Makiko Ichikawa et al. (8), serum miR-4297 expression of 170 glioma patients and 157 non-cancer subjects were obtained. Glioma patients were enrolled from those who underwent surgery for suspected brain or spinal tumors at the Department of Neurosurgery and Neuro-oncology of the National Cancer Center Hospital (NCCH) or who were referred to NCCH after undergoing surgery elsewhere from August 1, 2008, through May 1, 2016.

Furthermore, we conducted a prospective validation cohort during January 2017 and February 2021. Primary glioma patients who received the surgery in Neurosurgery Department at the First Affiliated Hospital of Fujian Medical University were enrolled. The last date of follow-up was 10th February 2021. Patients who met the following criteria were recruited: (1) age > 18 years; (2) gliomas diagnosis and grade criteria fulfilling the 2016 WHO Classification of Tumors of the Central Nervous System (1); (3) at first diagnosis with gliomas, namely without the history of any radiation, chemotherapy or resection. The exclusion criteria were as follows: (1) patients with concurrent chronic comorbidities (i.e., cardiovascular disease, renal dysfunction, autoimmune, and hematological diseases; (2) history of malignancy; (3) patients with local or systemic infection; (4) patients taking medications that related with an inflammatory condition. Participants were categorized as low-grade gliomas (LGGs) and high-grade gliomas (HGGs) according to the World Health Organization (WHO) grading criteria of primary brain tumors (1). Progression-free survival (PFS) time was defined as from the date of treatment to the date of progressive disease or death. The PFS time of patients without progression or lost follow-up were censored at the time of the last tumor evaluation. The study was approved by the Ethics Committee of the First Affiliated Hospital of Fujian Medical University. The research was carried out in accordance with the World Medical Association Declaration of Helsinki, and all subjects provided written informed consent.

### Data collection

Demographic characteristics and clinical features were collected from the medical records. Baseline clinical characteristics were extracted from medical records. The first laboratory measures before surgery were adopted. Neutrophil/lymphocyte ratio (NLR), platelet/lymphocyte ratio (PLR), and monocyte/lymphocyte ratio (MLR) were calculated as the ratios of the absolute counts of neutrophil, platelet, and monocyte to lymphocyte count, respectively. Systemic immune-inflammation index (SII) was derived from the following formula: (platelet count × NLR). Prognostic nutritional index (PNI) = albumin [g/L] + total lymphocyte count × 5.

### Blood biomarker measurements

We collected and analyzed overnight fasting blood samples at the first laboratory measurement before surgery. Hematological variables were determined using ADVIA 2120i automated analyzer (Siemens Healthcare Diagnostics, Deerfield, IL, USA). The levels of serum albumin and creatinine were determined using Siemens ADVIA 2400 automatic biochemical analyzer. Plasma fibrinogen levels were determined by the coagulation method (Sysmex CS-5100 Hemostasis System, Siemens). Within 4 h of sampling, serum samples were timely isolated by centrifugation method and transferred to RNase/ DNase-free tubes for further processing.

### MiR-4297 detection and quantification

RNAs were extracted from 200 μl serum samples by miRNA isolation kit (Tiangen Biotech CO., LTD., Beijing) and then stored at −80°C in RNase/DNase-free tubes. The RevertAid First Strand cDNA Synthesis Kit (Thermo Scientific Fermentas, USA) was applied to carry out the reverse transcription to prepare cDNA. The quantitative real-time PCR was performed using the ABI QuantStudio Dx real-time PCR instrument (Thermo Fisher Scientific, USA) according to the manufacturer's protocols. SYBR Green-based qPCR was performed in a 20 μl reaction system, including 2 μl of five-fold diluted cDNA.

MiR-4297 expression was normalized with U6 expression within each sample. The 2–^ΔΔ*CT*^ method was employed to analyze the relative expressions of miR-4297. Results with CT values >30 were considered unreliable and were excluded from the subsequent analysis. Specific primer sequences used are presented in Supplementary Table 1.

### Luciferase reporter gene assays

Human 293T cells were cultured in 24-well plates in a DMEM medium. Firefly and Renilla luciferase activities were measured using the dual-luciferase assay kit (Promega) according to the suppliers' specifications. The luciferase reporter construct containing the rs7896488 G allele is designated as pGL3-miR4297-MGMT3'-UTR-WT (P-G). The luciferase reporter constructs containing the site-specifically mutated rs7896488 A allele is designated as pGL3-miR4297-MGMT3′-UTR-MT(P-A). The luciferase reporter constructs containing P-G, P-A, and P-NC were transiently transfected into 293 T cells, respectively. Experiments were conducted 3 times, respectively, and three parallel samples were measured each time. Finally, data were expressed as the mean ± SEM. Luciferase activity was normalized to Renilla luciferase activity to correct for the differences in transfection efficiency.

### Genotype analysis

PolymiRTS Database 3.0 (https://compbio.uthsc.edu/miRSNP/) was employed to predict the variant rs7896488 in MGMT 3′UTR was located in the specific binding region of miR-4297. Thus, we isolated genomic DNA samples of 138 glioma patients and 327 healthy controls which were reported in our previous study (19) from the peripheral blood using a commercial Tiangen TIANamp Genomic DNA kit (Tiangen Biotech., Beijing, China). Additionally, we utilized KAPA Express Extract Kits (KAPA Biosystems, Wilmington, MA, USA) to isolate 12 genomic DNA samples of cases (12/150, 8%) from paraffin-embedded normal tissue adjacent to cancer specimens to increase the sample size of cases. The clinical characteristics of the subjects are shown in Supplementary Table 2. Genotypes of the candidate variant rs7896488 were determined by the Sequenom MassARRAY iPLEX platform (Sequenom Inc., San Diego, CA, USA). Genotyping was performed without knowledge of the case/control status of the subjects.

### Statistical analysis

Continuous variables were displayed as mean ± standard deviation (SD) or median (25th, 75th percentile). Categorical variables were shown as numbers and percentages. All data were checked for normality and homogeneity of variance by the Shapiro–Wilk test and Bartlett test. Bonferroni correction was used to adjust the *p*-value for multiple testing. Student's *t*-test and Mann–Whitney *U*-test were applied for comparisons of continuous variables between two groups, as appropriate. The sex-dependent associations of MPV, MGMG protein, and IDH mutation status with miR-4297 levels and FHGRS were evaluated by Spearman rank correlation analysis. Multivariate stepwise logistic regression analysis was carried out to investigate the independent variables for predicting high-grade gliomas. The multivariate logistic regression model was evaluated by the area under the receiver-operating curve (AU-ROC) analysis. PFS curves were plotted using the Kaplan-Meier method and compared by the log-rank test. Statistical analyses were performed in SPSS 17.0 (SPSS Inc., Chicago, IL, USA). All *p*-values given were 2-sided and a *p* < 0.05 was statistically significant.

## Results

### Baseline clinical characteristics and serum miR-4297 expression

As shown in Table 1, serum miR-4297 levels in glioma patients were remarkably reduced in age- and sex- matched case-control study (*p* < 0.001). Furthermore, a total of 114 glioma patients who received surgery in the Neurosurgery Department at the First Affiliated Hospital of Fujian Medical University from January 2017 to February 2021 were enrolled and followed up, including 66 males and 48 females. The mean age of all participants was 45.96 ± 12.22 years. There were 33 low-grade gliomas (LGGs), characterized by diffuse astrocytoma (21) and oligodendroglioma (12). A total of 81 high grade gliomas (HGGs) were listed as follows: anaplastic astrocytoma (13), anaplastic oligodendroglioma (9), and glioblastoma (59).

**Table 1:** Demographic data and clinical features of the subjects.

	**Glioma** **(*****N*** = **170)**	**Non-cancer controls** **(*****N*** = **157)**	***p-*** **value**
**GSE139031 dataset**			
Age (years, mean ± SD)	52.3 ± 17.45	55.18 ± 14.19	0.102
Male (%)	93 (54.7)	76 (48.4)	0.270
Serum MiR-4297	1.041 (0.221,2.022)	2.185 (0.828,2.935)	<0.001
	**Glioma patients** **(*N* = 114)**	**Serum miR-4297** **expression**	
**Validation cohort**			
Age (years, mean ± SD)	45.96 ± 12.22		0.048
<46.0	59 (51.8%)	0.612 (0.151, 1.576)	
≥46.0	55 (48.2%)	1.154 (0.245, 3.340)	
**Sex (%)**			0.035
Male	66 (57.9)	0.779 (0.173, 1.613)	
Female	48 (42.1)	1.392 (0.425, 3.194)	
**WHO grade (%)**			0.042
Low grade	33 (28.95)	0.521 (0.219, 1.325)	
WHO II	33 (28.95)		
High grade	81 (71.05)	1.082 (0.116, 3.481)	
WHO III	23 (28.39)		
WHO IV	58 (71.60)		
**Glioma subtypes, *N* (%)**			0.216
DA, IDH-mut	18 (15.79)	0.446 (0.251, 0.680)	
DA, IDH-wt	7 (6.14)	2.186 (0.196, 7.951)	
AA, IDH-mut	5 (4.38)	0.768 (0.200, 1.061)	
AA, IDH-wt	10 (8.77)	0.676 (0.800, 1.988)	
AOD, IDH-mut	7 (6.14)	1.230 (0.460, 2.694)	
OD, IDH-mut	8 (7.01)	1.593 (0.157, 2321)	
GBM, IDH-mut	10 (8.77)	0.521 (0.344, 0.783)	
GBM, IDH-wt	49 (42.98)	1.491 (0.400, 4.170)	
**Tumor locations (%)**			0.297
Hemisphere	102 (89.5)	0.853 (0.249, 2.312)	
Cerebellum	4 (3.5)	0.828 (0.2593, 2.635)	
Thalamus/hypothalamus	4 (3.5)	2.454 (0.3414, 5.756)	
Pons/medulla/brainstem	4 (3.5)	0.231 (0.121, 0.389)	
**Tumor size before RT (%)**			0.305
<6 cm	77 (67.5)	1.014 (0.250, 2.515)	
>6 cm	37 (32.5)	0.712 (0.179, 1.732)	
**MGMT promoter**			0.650
Methylation (%)	64 (56.1)	0.822 (0.254, 2.323)	
Unmethylation (%)	40 (35.1)	1.083 (1.821, 3.038)	
**IDH status, *n* (%)**			0.025
Mutation	47 (41.2)	0.669 (0.266, 1.593)	
Wild type	61 (53.5)	1.363 (0.278, 3.582)	
**1p/19q codeletion, *n* (%)**			0.771
Negative	52 (45.6)	0.797 (0.368, 2.347)	
positive	22 (19.3)	1.230 (0.187, 2.330)	
**Treatment (after surgery)**			
concurrent chemoradiotherapy	82 (71.9)		
Chemotherapy/radiotherapy	14 (12.3)		
none	18 (15.8)		

Data are expressed as mean ± SD, median (25th, 75th percentile) or number (%).

DA, diffuse astrocytoma; AA, anaplastic astrocytoma; AOD, anaplastic oligodendroglioma; OD, oligodendroglioma; GBM, glioblastoma; IDH, isocitrate dehydrogenase; RT, radiotherapy; MGMT, O 6- methylguanine DNA-methyltransferase; wt, wild type; mut, mutant.

We observed serum miR-4297 levels were obviously increased in the HGGs (*p* = 0.042). Besides, miR-4297 expressions in women participants were apparently higher than those in men (*p* = 0.035). However, significant differences in serum miR-4297 levels were not found when glioma patients were entirely categorized according to histology classifications. However, when compared to those in IDH-mt diffuse astrocytoma, serum miR-4297 levels were notably higher in IDH-wt GBMs (*p* = 0.020). In addition, although elevated levels of serum miR-4297 in IDH-wt GBM were observed as compared to those in IDH-mt GBMs, the comparison did not reach statistical significance (*p* = 0.068). Although serum miR-4297 was elevated for the glioma in the thalamus / hypothalamus, they did not statistically differentiate from tumor locations. The notable association of serum miR-4297 levels with glioma size before radiotherapy was not shown either.

### Sex-dependent association of miR-4297 levels with clinical features

**Figure 2:**
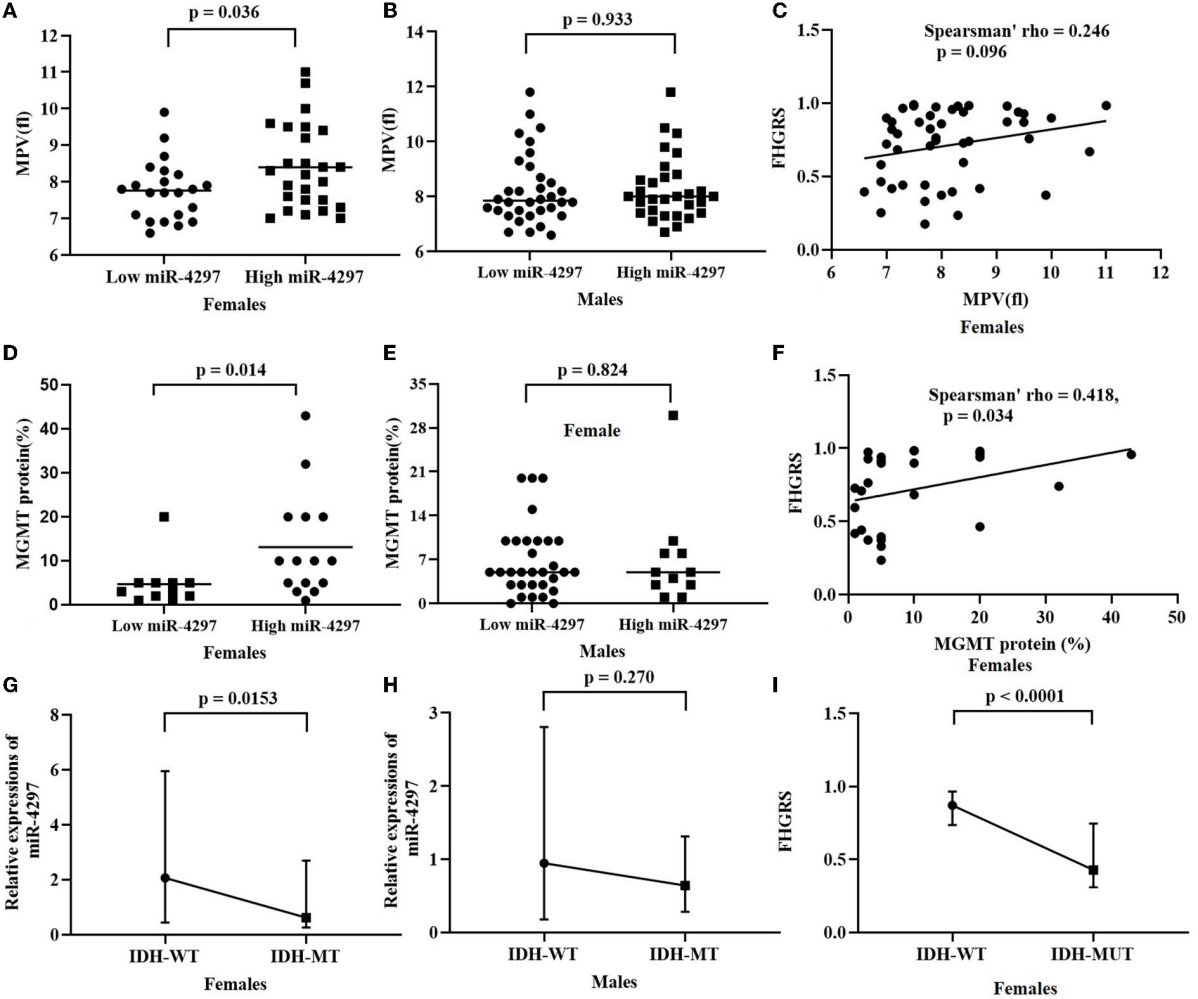
Sex-dependent analysis of serum miR-4297 and FHGRS. **(A)** Rather than FHGRS, statistically significant correlates are observed between miR-4297 levels and MPVs in female patients; **(B)** MGMT protein correlates with miR-4297 levels and FHGRS in females; **(C)** IDH-MT female patients have significantly decreased levels of miR-4297 and FHGRS. *MPV*, mean platelet volume*; MGMT*, O6-methylguanine-DNA methyltransferase; *WT*, wild type; *MT*, mutant type.

Based on the median levels of serum miR-4297, both female and male participants were, respectively, classified into two groups: miR-4297 high-expression group and miR-4297 low-expression group (Table 2). Clinical features, including routine molecular genetic parameters and immunochemistry indicators, and blood biomarkers were compared. Totally, isocitrate dehydrogenase (IDH) mutation tests were performed in 108 glioma specimens; 98 specimens underwent MGMT promoter methylation measurements; 74 specimens had 1p/19q codeletion analysis. Significantly high miR-4297 levels were prone to be observed in high-grade glioma females (*p* = 0.023), while there was no obvious tendency in males. Among women patients, serum miR-4297 expression statistically correlated with IDH mutation (*p* = 0.0153, Figure 2). Specifically, 54.2% of female patients in low—expression miR-4297 group had IDH 1/2 mutation, whereas only 20.8% of subjects in high - expression miR-4297 group were IDH1/2-mutant gliomas (Table 2). However, serum miR-4297 levels were not significantly associated with the status of methylated MGMT or 1p/19q codeletion in both sexes. As depicted in Figure 2, serum miR-4297 levels were positively associated with MGMT protein expressions in female glioma specimens (*p* = 0.014). Regarding routine hematological markers, we found a notable positive correlation between serum miR-4297 level and mean platelet volume (MPV) in female patients (*p* = 0.036). Additionally, there were no significant relationship between miR-4297 with plasma fibrinogen, serum albumin, or serum creatinine. On the other side, we did not find any statistical associations of miR-4297 with clinical features or hematological markers in glioma male patients (Table 2).

**Table 2:** Clinical parameters in miR-4297 low and high expression group by sex.

	**Male (*****n*** = **66)**		***p***	**Female (*****n*** = **48)**		***p***
	**Low miR-4297**	**High miR-4297**		**Low miR-4297**	**High miR-4297**	
Age (years, mean ± SD)	39.73 ± 15.52	46.58 ± 14.44	0.066	46.33 ± 10.82	46.17 ± 14.30	0.964
HGGs, *N* (%)	21 (63.6)	25 (75.8)	0.172	13 (54.2)	22 (91.7)	0.023
**Molecular genetic biomarkers**						
pMGMT–Me, *N* (%)	18 (60.0)	16 (55.1)	0.702	16 (69.6)	14 (63.6)	0.759
IDH-mutant, *N* (%)	17 (51.5)	12 (36.3)	0.277	13 (54.2)	5 (20.8)	0.015
1p/19q codeletion, *N* (%)	6 (25.0)	10 (45.0)	0.210	2 (8.33)	4 (16.7)	0.587
**Immunochemistry indicators**						
MGMT protein (%)	4.50 (1.75, 6.50)	5.00 (3.00, 9.00)	0.824	4.00 (2.00, 5.00)	10.00 (4.50, 20.00)	0.014
P53 (%)	17.50 (4.75, 80.00)	30.00 (6.50, 55.00)	0.811	30.00 (6.25, 78.75)	15.00 (4.50, 70.00)	0.843
Ki-67(%)	20.00 (6.00, 52.50)	20.00 (9.50, 35.00)	0.980	15.00 (5.00, 30.00)	17.50 (4.25, 42.50)	0.310
**Blood parameters**						
Fibrinogen (g/L)	3.17 ± 1.07	3.04 ± 1.13	0.638	2.93 ± 0.63	2.82 ± 1.061	0.674
Albumin (g/L)	43.16 ± 3.57	42.17 ± 3.51	0.285	41.47 ± 2.94	42.90 ± 5.53	0.294
Creatinine (μmol/L)	67.45 ± 9.09	67.97 ± 14.12	0.890	50.18 ± 7.00	48.09 ± 7.63	0.341
WBC ( ×10^9^/L)	7.60 ± 3.20	8.39 ± 3.08	0.324	7.34 ± 2.52	7.47 ± 2.93	0.874
M ( ×10^9^/L)	0.43 (0.31, 0.56)	0.39 (0.30, 0.48)	0.807	0.29 (0.26, 0.43)	0.43 (0.22, 0.53)	0.379
L ( ×10^9^/L)	1.65 ± 0.64	1.90 ± 0.68	0.123	1.78 ± 0.684	1.64 ± 0.548	0.472
Ne ( ×10^9^/L)	5.41 ± 3.10	5.70 ± 3.28	0.723	4.90 ± 2.40	5.29 ± 2.88	0.628
RBC ( ×10^12^/L)	4.91 ± 0.49	4.77 ± 0.33	0.173	4.39 ± 0.412	4.48 ± 0.484	0.488
HGB(g/L)	146.64 ± 15.46	146.97 ± 7.90	0.917	129.00 ± 16.28	131.58 ± 15.34	0.582
MCV (fl)	87.70 ± 7.90	89.76 ± 4.51	0.211	87.99 ± 7.66	87.51 ± 7.42	0.832
HCT	0.444 ± 0.008	0.428 ± 0.021	0.346	0.38 ± 0.046	0.39 ± 0.038	0.483
RDW (%)	13.34 ± 0.84	13.20 ± 0.96	0.528	13.95 ± 2.59	13.72 ± 1.10	0.233
MPV (fl)	8.24 ± 1.27	8.27 ± 1.16	0.933	7.76 ± 0.820	8.40 ± 1.15	0.036
NLR	2.14 (1.27, 2.53)	1.41 (1.12, 1.81)	0.300	2.79 (1.60, 3.48)	2.34 (1.42, 4.12)	0.817
PLR	111.17 (79.89, 165.14)	86.70 (63.53, 115.56)	0.087	141.31 (107.59, 200.35)	123.60 (98.81, 166.12)	0.852
SII	485.93 (276.64, 718.29)	306.84 (235.00, 446.37)	0.189	575.53 (448.34, 652.20)	591.26 (243.94, 591.26)	0.700
MLR	0.25 (0.20, 0.30)	0.18 (0.15, 0.24)	0.324	0.19 (0.16, 0.26)	0.21 (0.13, 0.27)	0.582
PLT/WBC	37.08 ± 11.75	32.02 ± 11.84	0.126	41.48 ± 19.47	36.61 ±17.62	0.378
PNI	50.19 ± 8.58	51.71 ± 4.35	0.391	48.47 ± 9.84	51.12 ± 6.65	0.286

Data are expressed as mean ± SD, median (25th, 75th percentile) or number (%).

HGG, high grade glioma; pMGMT-Me, O6-methylguanine-methyl-DNA-transferase promoter methylation; IDH, isocitrate dehydrogenase; Alb, albumin; HGB, hemoglobin; WBC, white blood cell; M, monocyte; L, lymphocyte; PLT, platelet; MCV, mean corpuscular volume; MPV, mean platelet volume; RDW, red blood distribution width; NLR, neutrophil-to-lymphocyte ratio; PLR, platelet-to-lymphocyte ratio; MLR, monocyte-to-lymphocyte ratio; SII, systemic immune-inflammation index; PNI, prognostic nutrition index.

### Predictive values of serum miR-4297 and FHGRS equation model in HGG females

**Figure 3:**
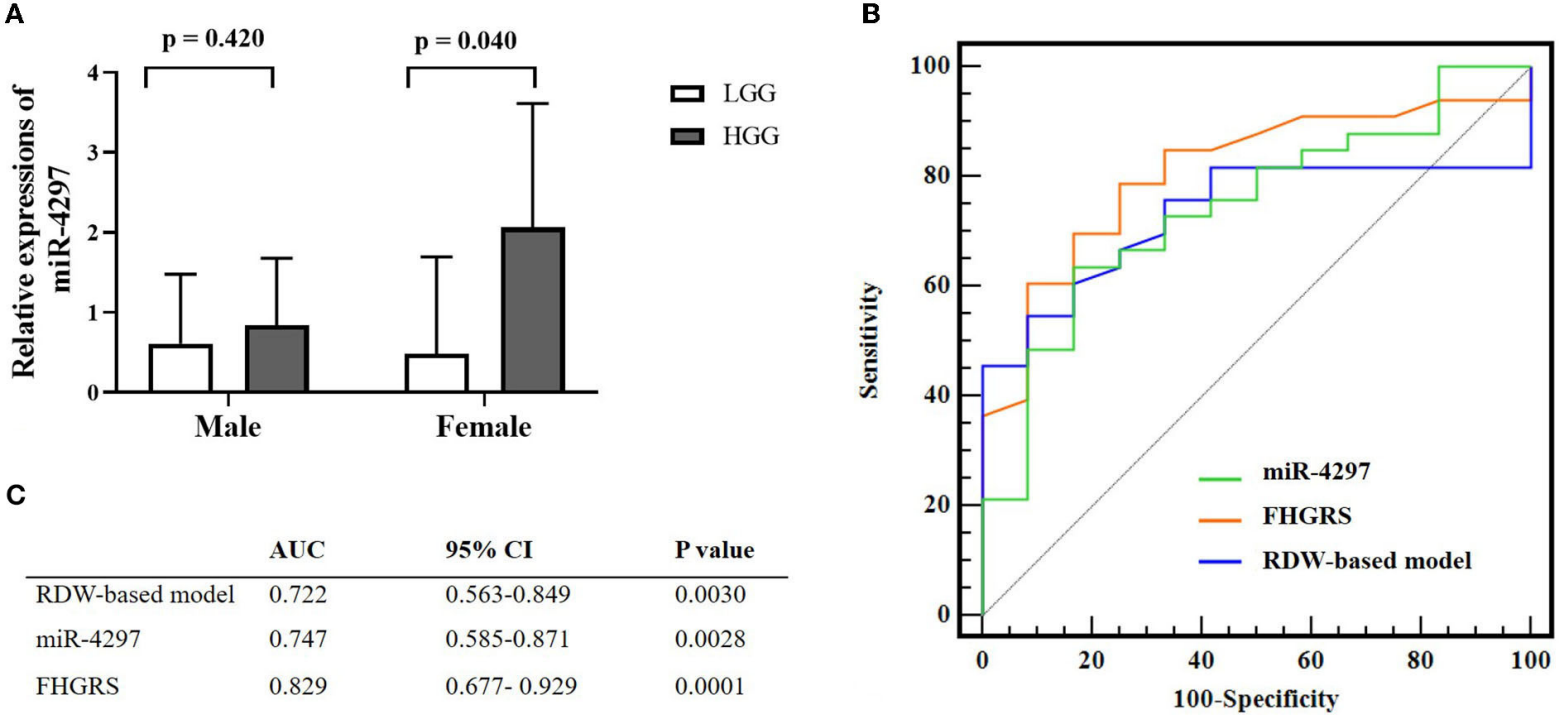
Pre-operative non-invasive biomarkers for female HGGs patients. **(A)** Sex-specific miR-4297 levels between LGGs and HGGs; **(B)** Comparison of miR-4297, FHGRS, and RDW-model for predicting HGGs in female patients by AU-ROC analysis. **(C)** AU-ROC values of RDW-based model, miR-4297 and FHGRS. *LGGs*, low-grade gliomas; *HGGs*, high-grade gliomas; *AU-ROC*, the areas under the receiver operating characteristic curves.

We conducted the univariate and multivariate regression analyses to investigate the age and blood parameters including miR-4297 expressions between LGGs and HGGs in the female subgroup. As a result, females in the HGGs group were older, with a remarkably elevated level of serum miR-4297 (*p* = 0.033 and 0.040, respectively). As depicted in Figure 3, AU-ROC analysis showed miR-4297 level > 1.421 generated a sensitivity of 63.64% and a specificity of 84.62% in predicting high-grade glioma in female subgroup (AUC = 0.747, 95% CI = 0.585–0.871, *p* = 0.0028). Furthermore, binary logistic regression analysis revealed that the age and serum miR-4297 were independent predictors for HGG females (odds ratios = 1.089 and 5.420, respectively, Table 3). After all, derived from the multivariate regression model, the risk score formula evaluating female HGGs (FHGRS) was established as follows:


$$\begin{array}{l} Female\;HGGs\;Risk\;Score\;\left(FHGRS\right)=\\ \frac{1}{1+e^{-\left(2.414^{*}miR-4297\#+0.094^{*}age-4.379\right)}} \end{array}$$

**Table 3:** Univariate and multivariate predictors for HGGs in females.

**Variables**	**Univariate**			**Multivariate**		
	**OR**	**95% CI**	***p-*** **value**	**OR**	**95% CI**	***p*** **-value**
Age	1.081	1.015–1.152	0.030	1.089	1.012–1.170	0.029
miR-4297 level	4.038	1.047–15.581	0.040	5.420	1.193–24.624	0.022

Variables with statistical significance in univariate analysis were entered into stepwise logistic regression analysis. OR, odds ratio.

(^#^ miR-4297 levels were transformed into the categorical variable according to the threshold of 1.421 by ROC analysis: miR-4297 level ≥1.421 represents 1; otherwise, 0).

AU-ROC analysis indicated FHGRS displayed a more powerful capability of predicting HGGs in females (AU-ROC = 0.829, 95% CI = 0.677–0.929, *p* = 0.0001). The equation FHGRS remarkably increased the sensitivity to 79.4% with a specificity of 71.4%. It exhibited an excellent positive predictive value of 87.1% and a modest negative predictive value of 58.8%. Furthermore, we extended our previously published RDW-based model to compare the diagnostic values of two models in our study (20). The RDW-based model exhibited an AU-ROC of 0.722 (95% CI: 0.563–0.849, *p* = 0.0030), By comparison, it was revealed that the AU-ROC of FHGRS was slightly higher than that of RDW-based model, although the difference between the two models was 0.107 (*p* = 0.150), as shown in Figure 3. Moreover, Spearman analysis revealed that FHGRS values in female patients were positive with MGMT protein expression (rho = 0.418, *p* = 0.034), and MPV (rho = 0.246, *p* = 0.096). Likewise, FHGRS values were remarkably higher in IDH-wt female gliomas.

### Sex-specific prognostic role of preoperative serum miR-4297

**Figure 4:**
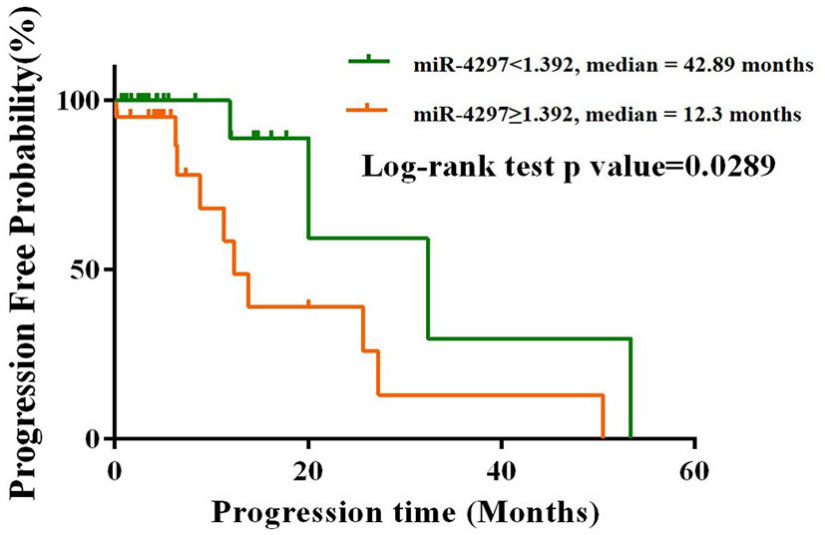
Kaplan–Meier progression-free survival curves for glioma female patients stratified by serum miR-4297 levels.

Totally, 114 glioma patients were followed up for nearly 50 months from January 2017 to February 2021. Among all the participants, 82 (71.9%) patients received the concurrent chemoradiotherapy after the surgery; 14 (12.3%) patients solely underwent chemotherapy or radiotherapy; the remaining 18 (15.8%) patients had no access to regular treatments after surgery. As depicted in Figure 4, the K-M analysis curves revealed that female patients with high levels of miR-4297 (≥1.329) had a median PFS of 12.3 months, as compared to 42.89 months for those with low levels of miR-4297 (log rank test: *p* = 0.0289). The hazard ratio (HR) for glioma recurrence with increased miR-4297 levels was 3.238 in females (95% CI:1.126–9.312). In contrast, serum miR-4297 expression was not associated with the risk for glioma relapse in the male subgroup.

### Dual luciferase reporter gene experiment

As depicted in Supplementary Figure 1, the dual-luciferase reporter gene experiment was performed to demonstrate the linkage between miR-4297 and MGMT. The mutant (mt) 3′- untranslated region (UTR) of MGMT was created through rs7896488 mutant allele (A allele) at the miR-4297-binding site. Compared with the miR-NC control group, the luciferase expression activity was significantly increased when the MGMT-3'UTR-wild type (wt) sequence containing the rs7896488 G allele was transfected with pre-mir-4297 overexpression vector (*p* < 0.001). On the contrary, the luciferase expression activity didn't show any obvious disparities when the MGMT-3'UTR-mutant type (mt) sequence containing the rs7896488 A allele transfected with pre-mir-4297 overexpression vector, as compared to the miR-NC control group.

### Genotype analysis for MGMT 3′UTR rs7896488 in patients and controls

The selected characteristics of enrolled subjects were summarized in Supplementary Table 2. The age and sex distributions between glioma patients and healthy controls showed no significant differences. Among them, 52 (37.7%) patients were low-grade glioma, while 72 (52.2%) patients were high-grade glioma. There were no clear grade records for the remaining 14 (10.1%) patients. Logistic regression analysis revealed that compared to participants carrying GG genotype, an increased risk for developing glioma was observed when the AG and AA genotypes were pooled for analysis, adjusted OR = 1.533 (1.044–2.370), *p* = 0.030 (Supplementary Table 3). Our findings indicated that the rs7896488 A allele may confer glioma susceptibility in an allele dose-dependent manner (test for trend *p* = 0.036). At a 7-year follow-up, we found glioma patients carrying the allele A (GA+AA) had a significantly shorter progression-free survival (29.6 vs. 47.3 months) in comparison to those with the allele G (GG), although the *p*-value showed no statistical significance (Supplementary Figure 2).

## Discussion

We provide the first evidence that preoperative serum miR-4297 probably serves as a valuable predictor for glioma grade and prognosis in a sex-specific manner. For decades, flourishing studies have demonstrated miRNAs get involved in the occurrence and development of glioma, and thereafter serum miRNAs could be surrogated as reliable biomarkers for glioma diagnosis and prognosis (8, 9). However, for the moment, there have been no widespread applications for potential miRNAs from the bench to the bedside. Hence, pinpointing the bottleneck for the clinical utility of miRNAs in glioma patients to facilitate personalized diagnosis and treatment has been challenging and deserves more effort. To the best of our knowledge, a variety of malignant tumors, including glioma, were often reported to happen in males with worse clinical outcomes. An increasing body of evidence from clinical studies has supported the sex-specific gene expression and pathway in glioma development (21–24). Male and female glioblastomas (GBMs) were found to exhibit sex-related responses to pharmacological or genetic inhibition (25). Undoubtedly, exploring sex-biased miRNAs holds some clues to identifying novel therapeutic approaches for glioma patients. HGGs are the most common malignant primary brain tumor in adults (1). In the current study, preoperative serum miR-4297 in glioma patients was remarkably elevated with increasing histopathological grades. However, when serum miR-4297 levels were segregated by sex, we found the statistical relationship between miR-4297 levels with HGGs was exclusively present in the female subgroup. Interestingly, the sex-dependent finding in the current study paralleled our previous study (20). Influenced by sex, it is well-recognized that red blood cell distribution (RDW) values are higher in females. In the previous independent cohort of 349 glioma patients, we found RDW levels were sex-dependent predictors for advanced glioma in female patients (20). Importantly, the RDW-based regression model was demonstrated to be promising for evaluating the prognosis of female gliomas. We validated the non-invasive RDW model in the current cohort, which was proved to exhibit consistent performance with the published result. After adjusting for clinical characteristics, hematological and biochemistry parameters by multivariate analysis, preoperative serum miR-4297, and age were identified as independent indicators of predicting advanced glioma for females. After all, the female high-grade glioma risk score (FHGRS) was derived from the miR-4297-based model, demonstrating a slightly robust capability of predicting the presence of HGGs (AU-ROC = 0.829 vs. 0.722) with the significant improvement of the sensitivity to 79.4% and the specificity to 71.4%, as compared to RDW-based model. Besides, the robust positive predictive capability of the FHGRS suggested that it would be helpful for confirming the new suspected cases before the invasive biopsy. Coincidentally, it was reported that the potential sex-biased expression of miRNAs may explain sex-specific cardiovascular pathophysiologies in women (26). Compared with males, differential expressions of miRNAs from myocardial cells for females may result in a sex-specific response of cardiomyocyte to injury or therapeutic inhibition. Furthermore, early identification of glioma recurrence is another challenge for the postoperative care of glioma patients. It has been well-recognized that the histologic changes alone could not provide accurate and timely information about the development and progress of the glioma (1, 27). We found serum miR-4297 levels showed no remarkable disparities among diffuse astrocytoma (DA), anaplastic astrocytoma (AA), and anaplastic oligodendroglioma (AOD), oligodendroglioma (OD), and glioblastoma (GBM). Nonetheless, considering molecular genetic markers, we observed both IDH-mt diffuse astrocytoma and IDH-mt glioblastomas had significantly decreased levels of serum miR-4297 in comparison with IDH-wt gliomas. It has been well-recognized that the IDH mutation signature is an emerging therapeutic target in glioma (1, 28). The present study also revealed that patients with IDH-mutant glioma characterized by a longer PFS time had more chances of a low expression of miR-4297. On the other side, MGMT, which is well-known as “a double sword,” repairs the DNA alkylation damage while contributing to TMZ chemotherapy resistance as well. High expression MGMT often represents a greater probability of relapse. Up to now, numerous clinical practices have argued that MGMT promoter methylation status could not well answer all puzzles for the treatment (29). Thus, MGMT protein expression has been revealed as the predictor of evaluating the response to TMZ (30, 31). Glioma patients with low expressions of MGMT protein were more likely to have favorable outcomes. In our study, there was no statistical correlation of serum miR-4297 levels with methylated MGMT status for all the patients; whereas miR-4297 levels positively correlated with MGMT protein expression on glioma specimen in the female subgroup. K-M PFS curves indicated that elevated miR-4297 levels above the threshold of 1.392 predicted a more than 3 times greater chance of glioma progression during the first 12.3 months after diagnosis in female patients. The results were consistent with the evident relationships of miR-4297 levels with MGMT protein expressions and IDH mutation status as well. As such, assessment of serum miR-4297 could unmask the independent prognostic significance, particularly in advance of glioma specimen molecular genetic tests and immunohistochemistry results. By contrast, glioma routine molecular genetic biomarkers, immunochemistry parameters, and hematological biomarkers did not show any obvious discrepancies when stratified by serum miR-4297 levels in the male subgroup. There have been documented several potential mechanisms of sexual dimorphism in microRNA expression (26, 32–34). On one hand, the estrogen (E2) gets involved in the transcription and processing of sex-biased miRNAs (35, 36). On the other hand, miRNAs distributed on X-chromosomes are approximately more than twice those in autosomes (33). Several X-chromosome-located miRNAs are expressed due to incomplete X-chromosome inactivation. MiR-4297, which is located in chromosome 10, probably regulates MGMT expression through binding to 3′ UTR of MGMT (Supplementary Figure 1). Although the underlying sex-bound association remained elusive, growing research has focused on that sex plays a vital role in glioma initiation and progression. The significantly larger proportion of MGMT promoter methylation and better outcomes for female patients has gained the attention of researchers (37). As compared to males, our present study presented the prominent increase of serum miR-4297 in parallel with MGMT protein on tumor tissues in females, which is probably attributed to sex disparities in MGMT expression in glioma. In addition, serum miR-4297 was positively correlated with mean platelet volume (MPV) in the female subgroup. As a routine hematological index of the average platelet size, it has been gradually recognized that MPV would reflect the inflammation response in different clinical settings more than the platelet function (38, 39). We have excluded patients with chronic inflammatory conditions from the current study so that the potential confounding factors on MPV were minimized. Nowadays, MPV has been accepted as a non-specific parameter for evaluating the severity of inflammation and the treatment therapy in malignant tumors (40). Meanwhile, it has become more popular to assess the potential link between platelet function and inflammation in the context of glioma (41, 42). Hence, elevated MPV values observed in the female high-expression miR-4297 group probably imply that miR-4297 fluctuations would reflect inflammatory responses to glioma development. On the other side, MPV reference intervals for males and females have been reported to display a statistical significance (43). Accordingly, sex hormone regulation is probably another plausible explanation for the positive association of MPV with miR-4297 in females since sex hormone regulation also played an indispensable part in the inflammation process (44). There are a few limitations to our study. Due to the high incidence in males for glioma, the present study recruited a relatively limited number of female patients. A small number of glioma patients had no access to more genetic tests of molecular markers because of a restricted extent of glioma resection. Thus, the statistical relationship of serum miR-4297 with molecular and immunohistochemical parameters may be subject to selection bias. Besides, significantly elevated levels of serum miR-4297 were found in gliomas located in the thalamus/hypothalamus, which accounted for only 3.5% of all the participants. However, nearly 90% gliomas were located at the cerebral hemisphere in the current study. Therefore, a relatively small proportion of gliomas located in the thalamus/hypothalamus could not reflect the actual disparities of miR-4297 levels among different locations. Therefore, larger well-designed studies will be needed to confirm our results. Nevertheless, the sex-dependent FHGRS signature of miR-4297 would be regarded as a reliable, cost-effective and non-invasive tool for neurosurgeons to precisely characterize female advanced gliomas, promptly evaluate the recurrent risk, and efficiently draw up optimal treatment strategies. The observation also pinpoints the profit of sex-specific analysis in non-coding RNA research, which could improve our understanding of sex-specific cellular mechanisms in glioma.

## Conclusions

In summary, the picture emerging from our study suggests that miR-4297 probably confers sexual dimorphism in glioma occurrence and development. In view of the observations at a molecular level, sex-specific therapeutic approaches would be tailored for the personalized management strategy.

## Data availability statement

The raw data supporting the conclusions of this article will be made available by the authors, without undue reservation.

## Ethics statement

The studies involving human participants were reviewed and approved by Ethics Committee of the First Affiliated Hospital of Fujian Medical University. The patients/participants provided their written informed consent to participate in this study.

## Author contributions

WX and LH contributed to the study conception and design. Data collection, analysis, and interpretation were performed by WX and BX. The first draft of the manuscript was written by WX. Study supervision was conducted by BY. All authors critically revised previous versions of the manuscript and approved the final manuscript.

## Funding

This work was sponsored by National Natural Science Foundation of China (Grant No. 81973118).

## Conflict of interest

The authors declare that the research was conducted in the absence of any commercial or financial relationships that could be construed as a potential conflict of interest.

## Publisher's note

All claims expressed in this article are solely those of the authors and do not necessarily represent those of their affiliated organizations, or those of the publisher, the editors and the reviewers. Any product that may be evaluated in this article, or claim that may be made by its manufacturer, is not guaranteed or endorsed by the publisher.
