# Supplementary material for: Serum microRNA-4297 is a sex-specific predictive biomarker of glioma grade and prognosis
Source: Front Neurol. 2022 Jul 27;13:888221. doi: 10.3389/fneur.2022.888221 (PMC9363699; doi:10.3389/fneur.2022.888221)

Supplementary Materials

Table S1 Primer sequences in this study

Primer name	Sequence (5’-3’)
MiR-4297 RT	GTCGTATCCAGTGCAGGGTCCGAGGTATTCGCACTGGATACGACACG GAA
MiR-4297 For	CGCGCGGTGTCTGTCC
MiR-4297 Rev	AGTGCAGGGTCCGAGGTATT
U6 RT	AACGCTTCACGAATTTGCGT
U6 For	CTCGCTTCGGCAGCACA
U6 Rev	AACGCTTCACGAATTTGCGT

RT reverse transcription, *For* forward, *Res* reverse

Table S2 Selected characteristics of cases and controls for rs7896488 genotype analysis

Characteristics	Cases (n=138)	Controls (n=327)	*p* value
Age, mean (SD)	44.4 (15.8)	48.4 (12.5)	0.095[a]
Sex, n (%)			0.887[b]
Male	90 (65.2)	211 (64.5)	
Female	48 (34.8)	116 (35.5)	
WHO grade, n (%)			
I	7 (5.1)		
II	45 (32.6)		
III	29 (21.0)		
IV	43 (31.2)		
Unknown	14 (10.1)		

[a] Wilcoxon rank sum test for comparison between two groups

[b] Two-sided χ^2 test for comparison between two groups

Table S3 Genotype frequencies of rs7896488 in patients and controls and its association with risk of glioma

Genotype	Patients (N = 138)	Controls (N = 327)	OR[a] (95% CI)	*p* value
GG	79 (57.25)	219 (67.80)	1.000 (reference)	
AG	53 (38.40)	94 (29.10)	1.563 (1.023-2.388)	0.039
AA	6 (4.35)	10 (3.10)	1.663 (0.585-4.726)	0.340
AG+AA	59 (42.75)	104 (32.20)	1.533 (1.044-2.370)	0.030
P_{trend}[b]				0.036

[a] ORs were calculated by logistic regression, adjusted for age and sex

[b] Tests for trend of odds were based on likelihood ratio tests assuming a multiplicative model

Supplementary FIGURE S1 miR-4297 directly targets MGMT3'UTR and upregulates MGMT expression

Human 293 T cells were transfected with luciferase reporter constructs containing pGL3-miR4297-3'-UTR-WT, pGL3-miR4297-UTR-MT, and pGL3-miR4297 NC, respectively. Experiments were conducted three times respectively and data were expressed as the mean ± SEM.

(A) miR-4297 directly targets MGMT3'UTR. The light blue highlight shows the binding site sequence. The red character represents MGMT gene rs7896488 (G>A), a polymorphism at the miR-4297 binding site; (B) miR-4297 probably promotes the luciferase activity of the wild-type MGMT 3'UTR-WT reporter gene vector containing the rs7896488 wild allele (G allele)

WT wild type, *MT* mutant type

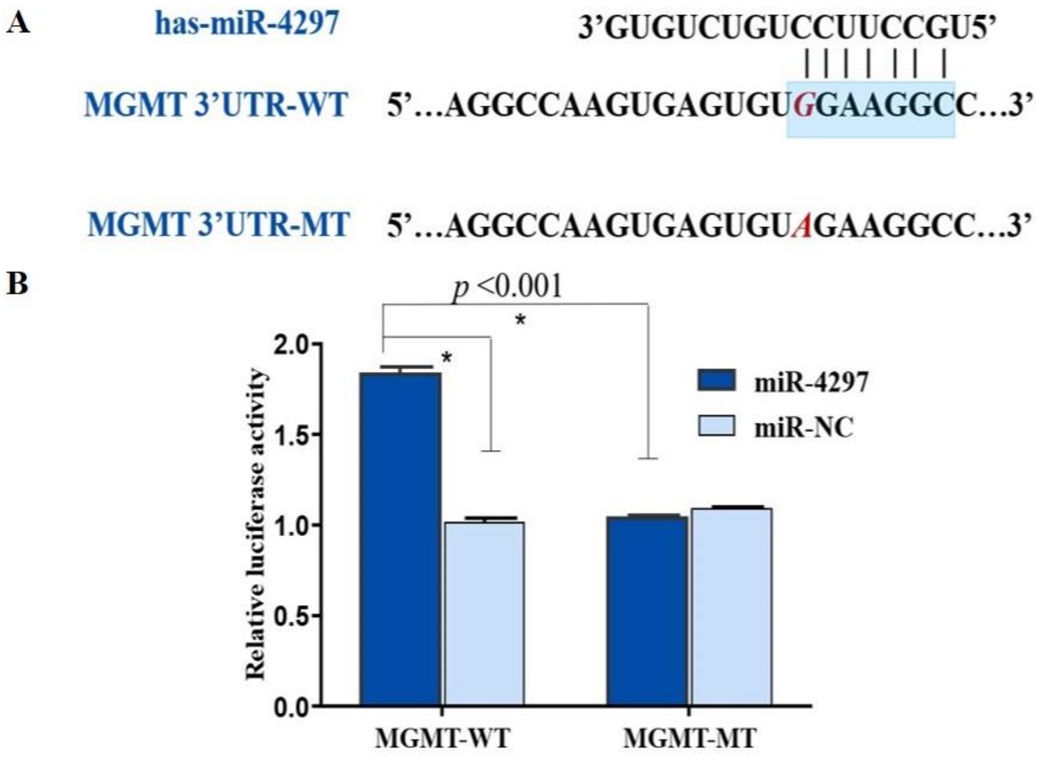

Supplementary FIGURE S2 Kaplan-Meier estimates of the PFS of glioma patients according to the binding site in *MGMT* 3'UTR rs7896488 genotypes. *PFS* progression-free survival

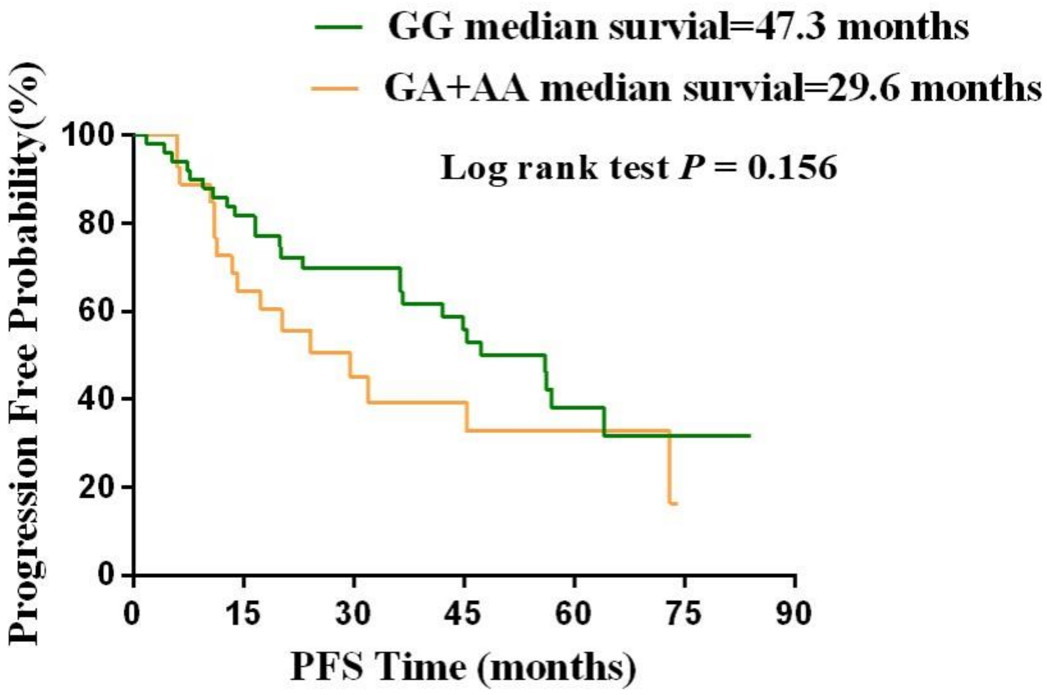

Supplement: Supplementary file 1 [file Data_Sheet_1.pdf]
